# Sex steroid hormones and behavior reveal seasonal reproduction in a resident fin whale population

**DOI:** 10.1093/conphys/coz059

**Published:** 2019-10-31

**Authors:** Erica Carone, Mario A Pardo, Shannon Atkinson, Kendall Mashburn, Héctor Pérez-Puig, Luis Enríquez-Paredes, Diane Gendron

**Affiliations:** 1 Centro Interdisciplinario de Ciencias Marinas, Instituto Politécnico Nacional, La Paz, Baja California Sur 23096, Mexico; 2 CONACYT - Centro de Investigación Científica y de Educación Superior de Ensenada, Unidad La Paz, La Paz, Baja California Sur 23050, Mexico; 3 College of Fisheries and Ocean Sciences, University of Alaska Fairbanks, Juneau, AK 99801, USA; 4 Kino Bay Center for Cultural and Ecological Studies, Prescott College, Bahía de Kino, Sonora 83340, Mexico; 5 Facultad de Ciencias Marinas, Universidad Autónoma de Baja California, Ensenada, Baja California 22800, Mexico

**Keywords:** Blubber, fin whale, progesterone, seasonal reproduction, testosterone

## Abstract

Fin whales in the Gulf of California constitute a resident population genetically isolated from the rest of the North Pacific Ocean. Its small population size and the scarce information available about its dynamics in a semi-enclosed sea underline the importance of conducting studies about its reproduction. Given the monsoonal regime that dominates the oceanographic habitat of this region, we hypothesized seasonality in the population’s reproductive activity. To test this, we validated and assayed testosterone and progesterone from blubber biopsies of free-ranging individuals. Lactating females exhibited low progesterone concentrations, whereas a group of females of unknown reproductive stage, but with extremely high progesterone concentrations, showed strong evidence of separation and were considered to be likely ovulating or pregnant. A seasonal model of testosterone concentrations showed a high peak during the late summer. This trend was supported by the first documentation of courtship events and by the recording of a female with high progesterone concentration during summer and re-sighted with a calf 1 year later. Therefore, the breeding in this resident population would be seasonal, as it is in migratory baleen whales, but occurring during the summer/autumn, which is the least productive season in the Gulf of California. Our study represents an important input to assist in future management policies of this protected population.

## Teaser

Fin whales in the Gulf of California constitute a resident population. To investigate its reproductive cycle, we measured the sex steroid hormones in their blubber. The temporal trend of testosterone, patterns in progesterone values, and courtship events suggest a seasonal cycle in which reproduction occurs during the least productive season.

## Introduction

Understanding the reproductive behavior of protected species is important for the development of effective conservation policies and management tools. It is especially crucial for small populations occupying restricted areas, such as islands, mountain lakes, or semi-enclosed seas, which make them more vulnerable to environmental changes and anthropogenic disturbances (Willi *et al.*, 2006). Although most baleen whales are known to regulate their breeding and feeding according to seasonal migrations between low and high latitude grounds (Mackintosh, 1965; Corkeron and Connor, 1999), the fin whale (*Balaenoptera physalus*) does not conform to this general pattern everywhere. The year-round occurrence of groups of this species, especially in mid-latitudes, has been reported (Silva *et al.*, 2013; Scales *et al.*, 2017), and the existence of small non-migratory populations is widely accepted (Fujino, 1960; Bérubé *et al.*, 1998).

**Figure 1:**
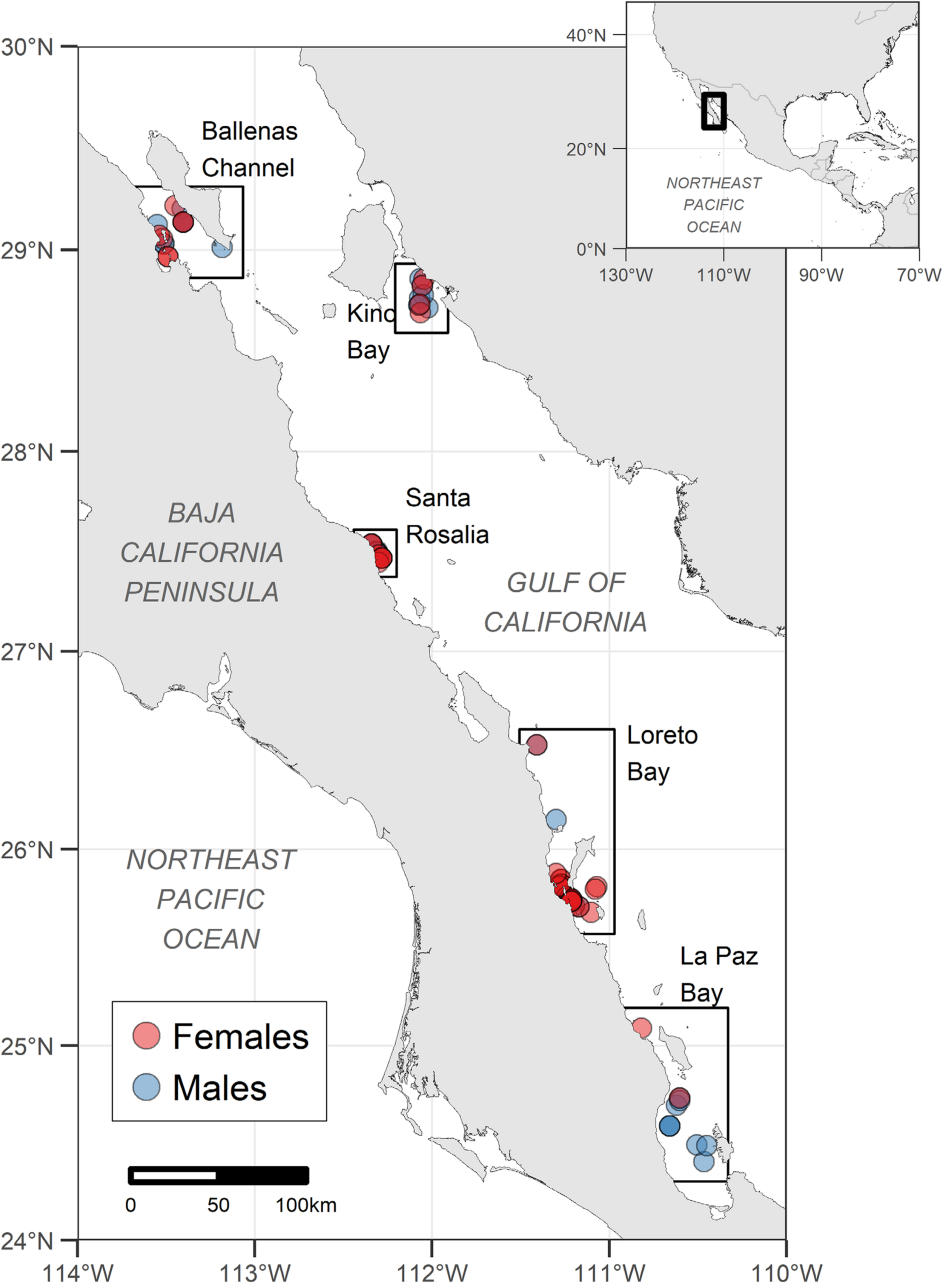
The Gulf of California in the Northeast Pacific Ocean. Colored dots show the geographic distribution of blubber biopsy samples from female (red) and male (blue) fin whales, used in this study. Each squared polygon was labeled according to the closest, most recognized geographical feature.

Genetic evidence shows that the fin whale population in the Gulf of California (Fig. 1) is unique and reproductively isolated (Bérubé *et al.*, 2002). A recent integrated study based on aerial surveys, genetic markers, and photographic capture/recapture produced a population abundance estimate of around 300 individuals [95% credible interval (CI) = 150–420] (Pardo *et al.*, 2016). Currently, there is no evidence that these animals move outside the Gulf of California, due to which the population is considered non-migratory. This resident strategy may suggest that the gulf fulfills the population’s requirements year-round, despite a strong monsoonal regime (Adams and Comrie, 2003) that favors high biological production during winter/spring, but more oligotrophic conditions during summer/autumn (Álvarez-Borrego and Lara-Lara, 1991). Although the habitat used by fin whales in the Gulf of California is not well known, it is possible that they aggregate during summer in some areas that remain productive due to upwelling or mixing, triggered by strong tidal regimes or other specific morphological features, capable of sustaining a rich macrofaunal community throughout the year (Brusca *et al.*, 2005).

In non-migratory mysticete populations, the primary driving forces on the reproductive strategies are still uncertain. In the Mediterranean Sea, the occurrence of fin whale newborns year-round, with a peak between September and January, suggests a breeding season not well defined for that resident population (Notarbartolo di Sciara *et al.*, 2003). Similarly, year-round calving and ovulation frequency on the inshore Bryde’s whales (*Balaenoptera edeni*) off South Africa proved that this resident population is an aseasonal breeder (Best, 2001). In contrast, the resident humpback whale (*Megaptera novaeangliae*) population in the Arabian Sea showed seasonal reproductive habits lasting from January to May (Mikhalev, 1997).

Given the strong restraints of cetacean studies in acquiring the basic knowledge of the physiological mechanisms driving reproduction, most of the current information comes from anatomical examination of gonads in commercially harvested, stranded, or by-catch animals (Lockyer, 1984), as well as from experiments in captivity (Daoquan *et al.*, 2006; Bergfelt *et al.*, 2011). In free-ranging cetaceans, most of the information about the life history parameters has been acquired through data collected from photo-identified animals (Agler *et al.*, 1993). Nevertheless, this fieldwork approach in areas not easily accessible may result in fragmentary information on individual sighting histories and their reproductive state. In this context, the measurement of hormones has become an important complementary tool for generating progressive and significant contributions to knowledge on baleen whale physiology (Hunt *et al.*, 2013).

There is strong evidence that mating behavior is regulated by changes in sex steroid hormone concentrations (Nelson *et al.*, 1990). Within these, progesterone and testosterone have been analyzed in different wildlife species (Schwarzenberger, 2007). Progesterone is mainly produced during the luteal phase of the estrous cycle and during pregnancy, in order to maintain quiescence of the uterus until the fetus is mature (Graham and Clarke, 1997), whereas testosterone concentrations respond to various factors, such as sexual maturation, competition, female estrous, and stress (Dixson and Anderson, 2004; Hansen, 2009). In cetaceans, steroid hormone concentrations have been quantified in serum, urine, saliva, milk, feces, ocular secretion, baleen, earplug, muscle, and blow (Yoshioka *et al.*, 1994; Amaral, 2010; Trumble *et al.*, 2013; Hunt *et al.*, 2017). Due to their lipophilic character (Prins *et al.*, 1996), these hormones have been detected also in blubber of several odontocetes and in humpback, bowhead (*Balaena mysticetus*), and minke whales (*Balaenoptera acutorostrata*) (Mansour *et al.*, 2002; Amaral, 2010; Kellar *et al.*, 2014; Cates *et al.*, 2019). The time lag between the production of sex steroids and their deposition in blubber and whether this tissue is representative of the current reproductive status of individuals are still uncertain. In the bowhead whale, there is a strong positive relationship between serum and blubber progesterone levels only when pregnant females are included (Kellar *et al.*, 2014). This means that even if blubber does not represent a target tissue or a vehicle of progesterone (Graham and Clarke, 1997), the magnitude and duration of its production during gestation are such that it is possible to detect its changes in blubber. Similarly, testosterone concentration in male blubber has produced useful data on seasonal reproductive patterns in humpback whales (Vu *et al.*, 2015) and short-beaked common dolphins (*Delphinus delphis*) (Kellar *et al.*, 2009).

Earlier studies of captured fin whales from the Atlantic and Pacific migratory populations showed that both females and males reach sexual maturity between 3 and 15 years old (Rice, 1963; Lockyer, 1972; Aguilar *et al.*, 1988). The mating season duration varies among populations, and conception probably occurs during winter (Mizroch *et al.*, 1984). Usually, females give birth to a single calf after a gestation period of about 11 months (Lockyer, 1984) and nurse them for 7–11 months before weaning (Mizroch *et al.*, 1984). Sex steroid hormones have been studied in the serum of fin whales caught in the North Atlantic during the summers of 1981–89 (Kjeld *et al.*, 1992). Based on concentrations found, it was possible to determine progesterone concentrations in pregnant females, as well as a temporal change in testosterone in males.

Although fin whales occur throughout the entire Gulf of California, there are some important gathering regions that have been identified (Pardo *et al.*, 2015; Jiménez López *et al.*, 2019) such as the Ballenas Channel, Kino Bay, Santa Rosalia, Loreto Bay, and La Paz Bay (Fig. 1). Nevertheless, the seasonality of such aggregations, as well as the reproductive behavior of the population, remains poorly understood. During 3 years of monthly surveys (1983–86) in the Ballenas Channel (Fig. 1), only 1% of sightings included mother/calf pairs (Tershy *et al.*, 1990). Calves can be seen year-round throughout the Gulf, similar to what has been observed for the resident population of the Mediterranean Sea (Notarbartolo di Sciara *et al.*, 2003). Despite that, the lack of information about the body length of calves observed has prevented the verification of seasonality in calving or mating events within the Gulf of California.

Given the strong seasonality of the oceanographic environment in the Gulf of California (Álvarez-Borrego and Lara-Lara, 1991), and considering the high energetic cost of reproduction for baleen whales (Lockyer and Brown, 1981), we hypothesized that this resident fin whale population has a seasonal reproductive cycle, which would be detectable from sexual hormone concentrations in blubber biopsies. Our objectives were as follows: (i) to quantify testosterone and progesterone concentrations in the blubber of fin whale individuals and relate them to their sighting histories, and (ii) to verify the existence of seasonal variations in the species’ testosterone concentrations. This is the first study based on reproductive hormones of free-ranging fin whales and constitutes a relevant advance in understanding this population’s dynamics in the Gulf of California.

## Materials and methods

### Sample collection

Samples and sighting data were collected under permits issued by “Secretaría de Medio Ambiente y Recursos Naturales” (SGPA/DGVS 08021/06, 00506/08, 09760/08, 01110/15, 00255/16, and 00987/17, issued to D. Gendron, and SGPA/DGVS//036624/17 and 002162/18, issued to M.A. Pardo; CITES export permits: MX 88860) and complied with the criteria of the Institutional Animal Care and Use Committee of the University of Alaska. We collected 84 skin/blubber biopsies from 34 females (four with more than one sample) and 44 males (one with two samples) of fin whales, spanning 2007–09 (*n* = 33) and 2015–17 (*n* = 51) in the following five main aggregation regions in the Gulf of California: La Paz Bay, Loreto Bay, Santa Rosalia, Kino Bay, and Ballenas Channel (Fig. 1). All biopsies were collected in accordance with the standard protocol (Costa-Urrutia *et al.*, 2013), using modified crossbow arrows, shot into the animal’s mid-lateral region, and stored in liquid nitrogen before being processed.

### Whale identification

The sex of each sampled animal was determined using skin (Bérubé and Palsbøll, 1996). Fin whales were identified individually using photographs of the dorsal fin profile and presence of permanent scars (Agler *et al.*, 1990). Adult females recorded with a calf were assumed to be lactating (*n* = 3), whereas the rest were considered as in an unknown reproductive stage. All males were considered as animals of unknown reproductive stage.

### Hormone extraction and quantification

Hormone extractions from blubber were carried out using the methods described by Mansour *et al.* (2002) with modifications. Approximately 0.06–0.15 g of blubber sample was crushed manually with 500 μL of ethanol. After centrifugation (2500 rpm for 15 min; Beckman Coulter GS-6R Centrifuge), the supernatant was collected, and the pellet was re-suspended in 500 μL of ethanol. Once dried, 2 mL of a 4:1 mixture of acetone and ethanol were added to the samples. Then, samples were mixed with a vortex (1 min; VWR Vortex-Genie 2), centrifuged (2500 rpm for 15 min), and evaporated. The same procedure was done first with 1 mL of ether and then with 2 mL of a 1:1 mixture of hexane and acetonitrile, twice. The final extracts were then frozen at −20°C until analyzed. Prior to the final analyses, the samples were re-dissolved in methanol. Progesterone for females and testosterone for males were quantified using commercially available enzyme immunoassay kits from Arbor Assays (catalogue #K025-H1; #K032-H1). Manufacturer cross-reactivity of progesterone assay with other steroids was as follow: 172% 3ß-hydroxy-progesterone, 188% 3a-hydroxy-progesterone, 2.7% 11ß-hydroxy-progesterone, 147% 11a-hydroxy-progesterone, 7.0% 5a-dihydroprogesterone, 5.9% pregnenolone, and <0.1% corticosterone and androstenedione. As for testosterone assay, the cross-reactivity was 100% with testosterone and 56.8% 5a-dihydrotestosterone, 0.27% androstendione, 0.04% androsterone and Dehydroepiandrosterone (DHEA), 0.03% cholesterol, 0.02% 17ß-estradiol, and <0.02% progesterone, pregnenolone, hydrocortisone, cholic acid, and cholic derivatives. All samples were processed and quantified in duplicate and were rerun if the coefficient of variation (CV) was higher than 10%. The maximum assay range allowed was between 50 and 3200 pg mL^−1^. If the concentration exceeded this range, the sample was rerun at varying dilutions. To validate the assays, we employed parallelism and accuracy tests in a pooled sample (derived by combining equal volumes of all rinse samples), done by randomly combining 20 samples for testosterone and 18 for progesterone. Parallelism test was based on the simultaneous run of pooled samples with the standard curve and the determination of parallelism between these two curves. The accuracy test determined the level at which the measured hormone concentration matched the concentration of the standard hormone added to the sample pool. This test allows the detection of the potential interference from other metabolites in the samples and is expressed as a linear regression of the measured hormone as a function to the standard. A slope much higher or much lower than one represents an over- or under-estimation of the hormone, respectively.

## Data analysis

The first step was to estimate the probability distributions of both hormones to obtain their ranges and central tendencies. A preliminary diagnosis of the progesterone’s frequency distribution (see Supplementary Text 1) showed a possible bimodal pattern in the logarithmic scale, with frequent extreme high values. Therefore, we performed a Bayesian mixture model of normal distributions on the progesterone observations (Marin *et al.*, 2005; Dahl, 2006), which allowed us to evaluate if there was evidence for such bimodal structure and to estimate the probability that the progesterone values come from each of the clusters detected by the model. For the testosterone, whose frequency distribution did not show apparent bimodal structure (see Supplementary Text 1), we made a simple Bayesian estimation of the mean (Gelman *et al.*, 2013). Both models were based on logarithmic likelihoods of the hormone concentrations.

In order to address the potential seasonality of fin whale reproductive cycle in the Gulf of California, we stated the testosterone concentrations as a sinusoidal function of the day of the year, whose parameter probabilities were estimated through a Bayesian regression analysis (Gelman *et al.*, 2013). A log-normal likelihood was stated for the testosterone observations, with uninformative priors. Given the limited amount of observations throughout the year cycle, we decided to complete the yearly series by replicating the observations made during winter as the first part of a hypothetical second year. This was based on the idea that it is not possible that very high values of testosterone maintain indefinitely and, because the predominantly low concentrations observed during the first part of the year suggest that it was a transition period.

**Table 1:** Posterior distribution summary of progesterone and testosterone means ($\mu$, ng g^−1^) in blubber of fin whales from the Gulf of California.

		Mean	SD	2.5%	25.0%	50.0%	75.0%	97.5%	$\hat{R}$	*N*_eff_ (%)
Progesterone										
	${\mu}_{\mathrm{Cluster}\ \mathrm{A}}$	1.58	1.30	0.97	1.33	1.56	1.84	2.76	1.00	92.86
	${\mu}_{\mathrm{Cluster}\ \mathrm{B}}$	26.05	1.66	8.55	19.43	27.12	36.24	64.78	1.00	100
Testosterone										
	$\mu$	0.88	1.23	0.59	0.77	0.88	1.01	1.32	1.00	65

A Gelman–Rubin convergence statistic ($\hat{R}$) close to one indicates good convergence of chains. The *N*_eff_ is the effective sample size as the percentage of the total iterations retained from all chains

**Figure 2:**
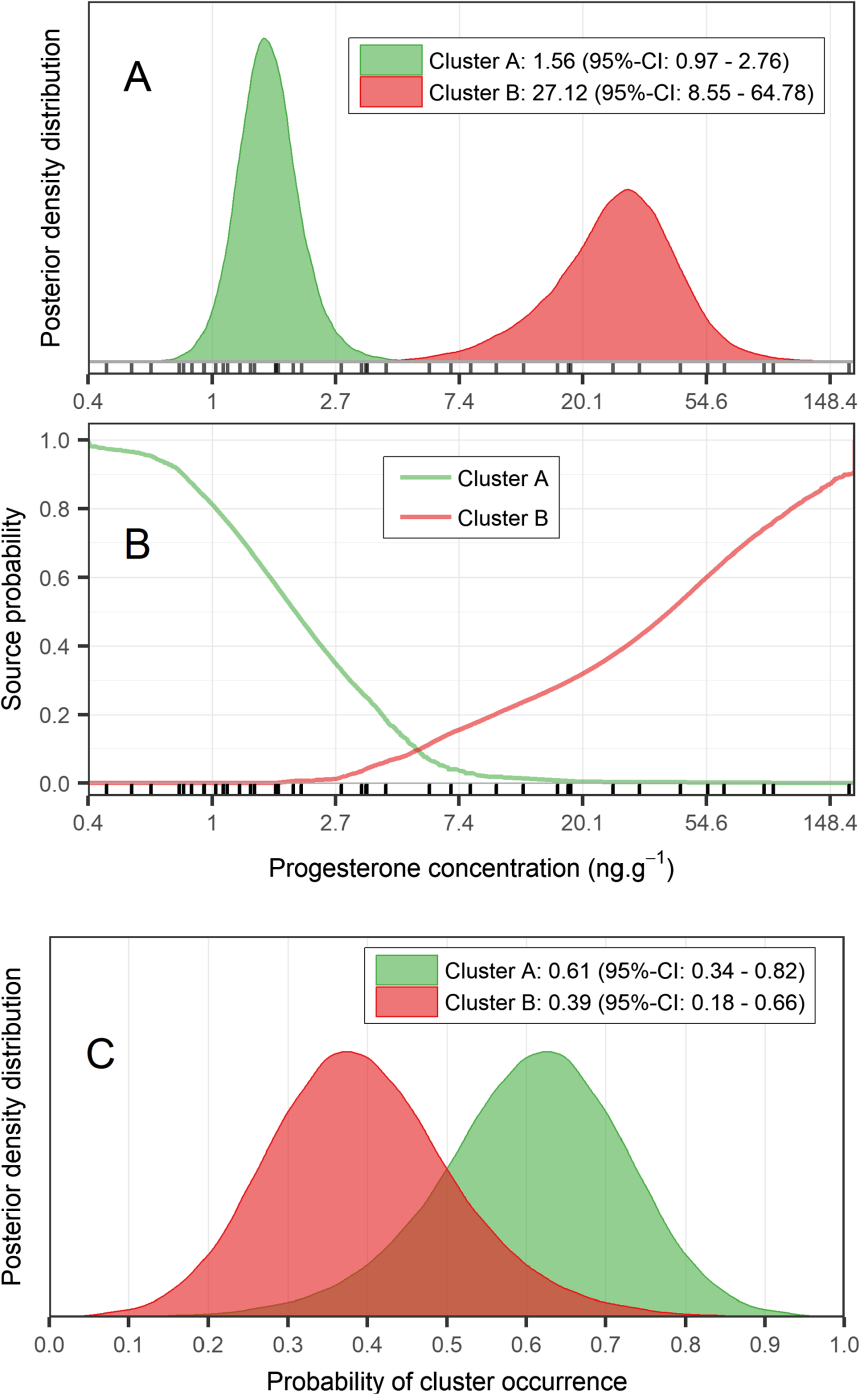
**Panel A**. Posterior probability distributions of progesterone concentrations (logarithmic scale) for the two clusters estimated by the Bayesian mixture model. **Panel B.** Posterior probability that any value within the observed range of concentrations comes from either Cluster A or Cluster B. **Panel C.** Posterior probability of cluster occurrence for each cluster.

**Figure 3:**
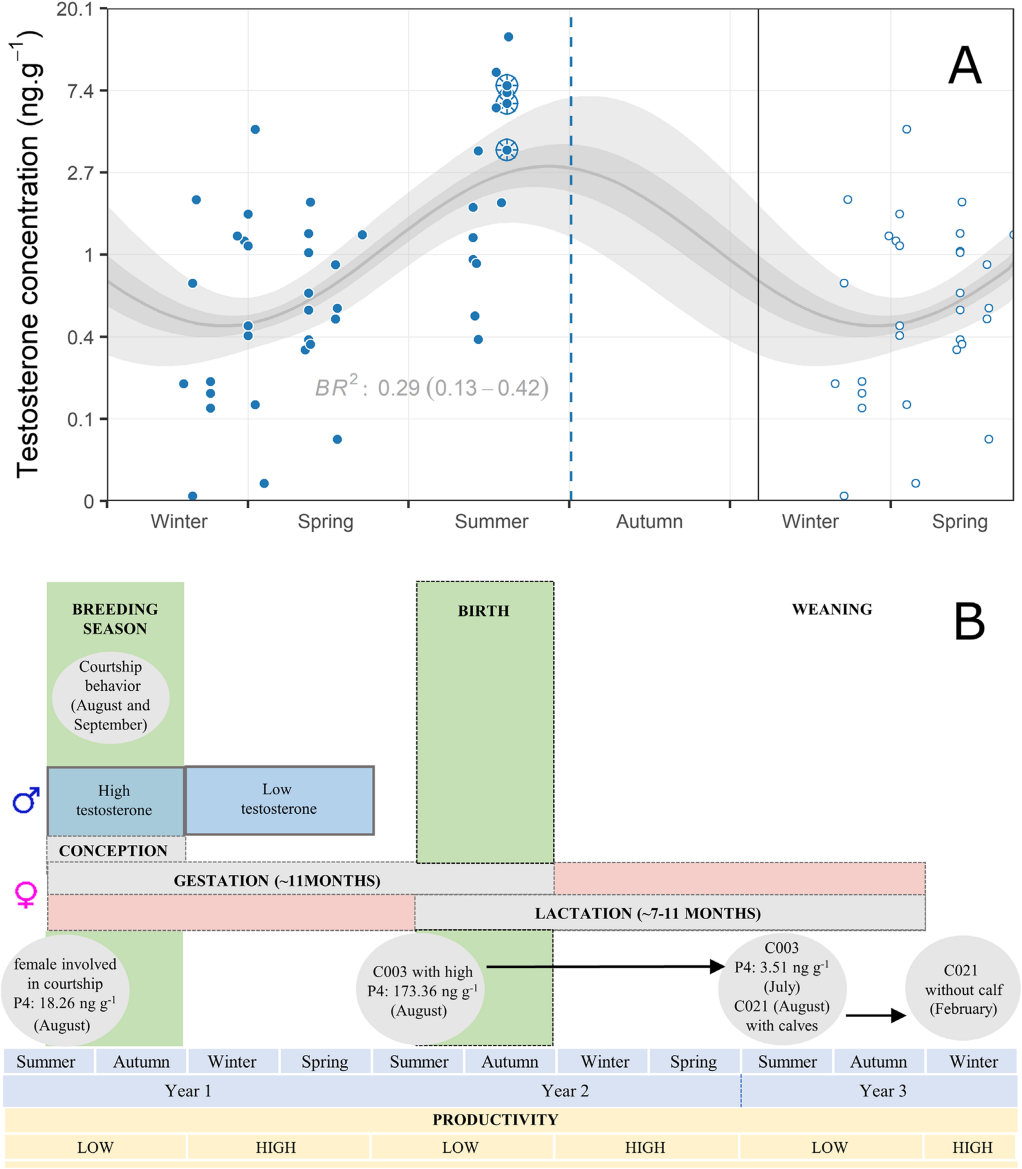
**Panel A.** Mean seasonal trend (gray sinusoidal curve) of testosterone concentrations in fin whale blubber biopsies in the Gulf of California, spanning 2007–09 and 2015–17. Shaded areas represent the 75% (dark) and 95% (light) credible intervals of the prediction. The mean BR^2^ with its 95% credible interval are shown. The Y-axis is in logarithmic scale. Color-filled dots represent the original observations and the empty dots represent the repetitions to complete the yearly series (see Materials and Methods). Observations surrounded by sliced circles represent animals involved in the first courtship event (2015). The vertical blue dashed line shows the day of the second courtship (2018; one female followed by two competing males, of unknown hormone concentrations). **Panel B**. Schematic reproductive cycle of the resident fin whale population of the Gulf of California, based on the seasonal hormone analysis of this study, the sighting histories of pregnant, lactating females and the female involved in the courtship event [with concentration of progesterone (P4)], weaning, and courtship events observed (gray circles). Black dashed lines represent a period of uncertainty during which each event is proposed.

All Bayesian analyses were performed within the Just Another Gibbs Sampler (JAGS) language (Plummer, 2003) to sample from the posterior distributions of the stochastic parameters through a Markov Chain Monte Carlo procedure. We ran 1 million iterations in three independent chains, keeping one of every 20 iterations to avoid autocorrelation of chain states, and discarding an adaptation (i.e. burn-in) phase of the first 10% of iterations per chain. The final sample size for each posterior distribution was of 135 000 iterations. All data processing, analyses, and graphical representations were performed in R (R Development Core Team, 2019). The algebraic details of the models and the JAGS codes are provided in Supplementary Text 1.

## Results

### Assay validation

Progesterone and testosterone assays were successfully validated. Serial dilutions of pooled extracts showed parallelism with the standard curves of progesterone and testosterone (see Supplementary Fig. 1). The slopes of the regressions between added and measured masses were close to a hypothetical 1:1 ratio, which suggests that the method was highly accurate in measuring the hormone concentrations from the samples. The slope was 0.83 for progesterone (95% CI = 0.78–0.87) and 0.83 for testosterone (95% CI = 0.76–0.9). Additionally, the high Bayesian *R*^2^ (BR^2^) suggests strong linearity between control and blubber extracts, which indicates that the assay was measuring primarily the same antigen ins both groups. The BR^2^ reached 0.998 in progesterone (95% CI = 0.994–0.999) and 0.997 in testosterone (95% CI = 0.985–0.997) (see Supplementary Fig. 2). The recovery efficiency was 111% (CV = 21.73%) for progesterone and 91.71% (CV = 23.91%) for testosterone.

### Progesterone and testosterone concentrations

The results of the mixture model of normal distributions strongly supported a bimodal structure in the progesterone concentrations. The first normal distribution of the mixture corresponded to the lowest values, with a median of 1.56 ng g^−1^ (95% CI = 0.97–2.76), whereas the distribution of the higher values had a median of 27.12 ng g^−1^ (95% CI = 8.55–64.78) (Table 1), with a slightly broader posterior probability (Fig. 2). The overlap between the curves was barely noticeable, limited to the tails of the distributions, and beyond the extreme limits of the 95% credible intervals of both clusters (Fig. 2). This separation was confirmed by the probabilities of cluster source for the range of the data (Fig. 2), which showed that the overlap only occurs at intermediate values, with probabilities less than 0.1. At that point, the probability that lower values come from cluster B is virtually 0 and vice versa. The results show that 61% (95% CI = 34–82) of the females were likely to come from cluster A (lower values) and 39% (95% CI = 18–66) from cluster B (higher values) (Fig. 2; see Supplementary Text 1). The testosterone exhibited a median of 0.88 ng g^−1^ (95% CI = 0.59–1.32) (Table 1; see Supplementary Table 1).

**Figure 4:**
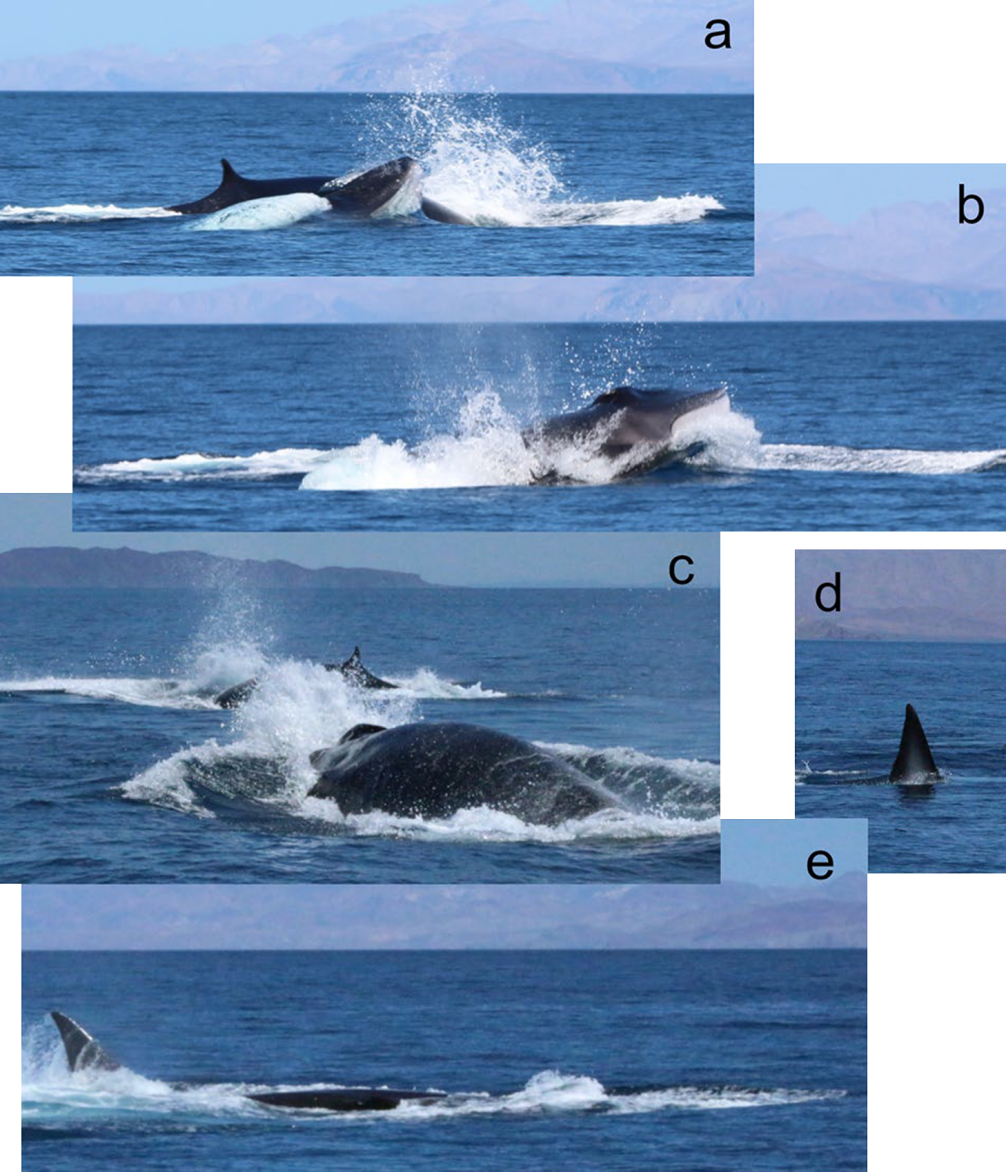
Two fin whale males during a presumed courtship display for a female (not shown) that always leaded their main path. They made fast and strong breaching displays very close to each other (a). Many times, the entire head was visible above the surface (b). They often irrupted each other’s path (c), and rolled on their longitudinal axes, showing half of the fluke (d and e) and a flipper out of the water. Photographs by G. Busquets-Vass (all rights granted).

### Reproductive seasonality

The seasonal model of testosterone concentrations showed a well-defined predicted high peak during late summer (August) and lower concentrations during late winter (February/March) (Fig. 3, panel A). The probability of positive amplitude in the sinusoidal curve was 100%, indicating high certainty on the curve trend. The variability explained by the model was relatively low (BR^2^ = 0.29; 95% CI = 0.13–0.42), but expected, since our model could not take into account inter-individual variability, and there was lack of observations during parts of the cycle. The information associated with the sightings of biopsied fin whales allowed us to detect some cases that suggested a seasonal pattern in the reproductive cycle of the population and confirmed the results of the seasonal model of testosterone concentrations. The female C003 was observed during summer (August 2015) in the Ballenas Channel (Fig. 1) with the highest progesterone concentration of all females biopsied (173.36 ng g^−1^). The same female was observed twice the next summer (July 2016) in Loreto Bay accompanied by a calf, presumably lactating, with a low progesterone concentration in blubber (3.51 ng g^−1^). Another female (C021) was observed during summer (August 2015) in the Ballenas Channel with a calf (a biopsy could not be collected). Almost 6 months later, during winter (February 2016), it was re-sighted and biopsied in Kino Bay without the calf, exhibiting a low progesterone concentration (0.76 ng g^−1^), which suggests that weaning probably occurred before winter. Finally, in the Ballenas Channel, our team witnessed two courtship events during summer, never described before for this species. On 12 August 2015, an individual was followed by three other fin whales at an anomalous highly sustained speed. The individuals of the group were seen at least twice side-lunging and obstructing each other’s path. Fortunately, we were able to collect biopsies from the four animals, and genetic analysis confirmed that the leading animal was a female exhibiting a medium progesterone concentration of 18.26 ng g^−1^ (cluster B; Fig. 2), whereas the other three animals were males with high testosterone concentrations (7.86, 6.32, and 3.58 ng g^−1^), above the highest limit of the 95% CI of the testosterone posterior probability. Finally, on 17 September 2018, we observed another courtship event between one female and two males (sex genetically confirmed), in the same area. As with the previous event, males were chasing the female, breaching alternately, and obstructing each other’s path (Fig. 4a–c; see Supplementary Video 1). On numerous occasions, both males turned on their longitudinal axes while surfacing, showing one of their flippers and half of the fluke (Fig. 4d and e).

## Discussion

To our knowledge, this is the first study quantifying progesterone and testosterone in free-ranging fin whales. The use of blubber, through non-lethal sampling techniques, allows the assessment of reproductive status at the population level, rather than only of hunted or sick individuals. Nevertheless, steroid hormones pathway in blubber remains unexplored. Due to their lipophilic properties, these hormones enter from capillaries into the fat cells via diffusion (Deslypere *et al.*, 1985), but there is no information about their accumulation or enzyme action on them once in the blubber. Neither testosterone (Kellar *et al.*, 2009) nor progesterone (Kellar *et al.*, 2006) are significantly different through the blubber in several species of delphinids, but in beluga whales (*Delphinapterus leucas*), cortisol concentration increased with blubber depth, with highest concentrations in the inner layer (Trana *et al.*, 2015). Despite the valuable information provided by these studies, they were carried out on odontocetes and there is still a lack of knowledge about mysticetes. Since blubber is a complex matrix characterized by different metabolic activity along its thickness, fatty acids, contaminant concentrations, and adipocyte size have been found to vary (Ackman *et al.*, 1975; Iverson, 2009; Waugh *et al.*, 2013). Thus, due to the greater blubber thickness of large whales, a different concentration of steroid hormones through the tissue can be expected. For this reason, to obtain comparable results in steroid analyses, it may be preferable to use the same biopsy subsample layer. Furthermore, as a result of these differences among species and the lack of knowledge of some physiological mechanism, it is crucial to validate the technique for measuring any hormone for each new species considered. Although knowledge of fin whale physiology is still limited, our validation was successfully carried out (see Supplementary Fig. 1; Fig. 2), and its results strongly support the use of blubber biopsies of fin whales to investigate reproductive aspects of the population and to detect long-term changes.

The lack of long sighting histories for most of the individuals sampled prevented us from determining hormone concentrations according to age classes. The highest progesterone concentration observed in this study (in summer) belonged to a female for which pregnancy was confirmed by its re-sighting a year later accompanied with a calf. This constitutes the first quantification of blubber progesterone in a pregnant fin whale. The wide range of progesterone values observed in this study (Fig. 2; see Supplementary Table 1) is in agreement with the high variability found in North Atlantic fin whale serum (Kjeld *et al.*, 1992) and in blubber of other cetaceans (Mansour *et al.*, 2002; Pérez *et al.*, 2011).

The presence of a group of females with high progesterone concentrations (cluster B, Fig. 2) may be explained by the luteal phase of the ovarian cycle or by the gestation. In this sense, the progesterone concentration of the female involved in the first courtship (2015) would reinforce the existence of this sexually active group of females (cluster B) in our mixture model. The intermedium progesterone concentration, close to the lower limit of the cluster B, could indicate that this female was in estrous (ovulating period) or recently conceived from a previous mating event during the same season. Nevertheless, it is still unknown which phase of the ovarian cycle may be detected through progesterone in large whales. Kjeld (1992), based on progesterone values in serum, identified the following three groups of females in the North Atlantic fin whale: (i) with low progesterone values (young sexually immature females); (ii) with intermediate values; and (iii) with high values (pregnant females). Due to the similar length and age with the first group, the author suggests that the second group represents mainly females that have recently maturated and in which an ovulation or a recent conception cannot be discarded. By contrast, blubber is considered a matrix that represents only long-term changes (Kellar *et al.*, 2014). Thus, considering that progesterone levels fall after ovulation if conception does not occur, it is likely that blubber reflects only pregnancy, which would explain the clearly distinction of two groups in our study, in accordance with previous studies on the same tissue (Mansour *et al.*, 2002; Kellar *et al.*, 2006) and in discordance with the serum results of Kjeld (1992).

The wide range of values in cluster B also may be linked to a high variability in the timing of conception during the breeding season, or simply to its lower sample size. In various mammals, progesterone serum levels gradually rise through the gestation (Bedford *et al.*, 1972; Boyd *et al.*, 1993). Nevertheless, the progesterone trend and whether its concentration in blubber reflects different gestation states are still unclear in cetaceans. Bottlenose dolphins (*Tursiops truncatus*) and killer whales (*Orcinus orca*) showed higher progesterone concentrations in serum during early and mid-pregnancy compared to late pregnancy (Katsumata *et al.*, 2006; Bergfelt *et al.*, 2011). In blubber of three species of dolphins, no difference was found in progesterone concentrations with respect to fetal length (Kellar *et al.*, 2006). In mysticetes, however, blubber is highly variable in thickness and composition, and no studies have investigated progesterone throughout the entire gestational period.

Even if the physiological processes that drive progesterone production in cetaceans are not clear, we hypothesized, based on studies in captive odontocetes (Bergfelt *et al.*, 2011), a rapid decrease of the hormone immediately before and during parturition. This was confirmed by the low progesterone concentrations found in lactating females in this study. Furthermore, since fin whales show long calving intervals of at least 2 years (Mizroch *et al.*, 1984), we assumed that progesterone production, in resting females, was similar to that of lactating females, according to the pattern reported previously in blue whale (*Balaenoptera musculus*) feces (Valenzuela *et al.*, 2018) and blubber (Atkinson *et al.*, 2019). These animals would be represented by the cluster A (Fig. 2) identified in this study.

The high testosterone concentrations we observed in late summer coincide with the pattern found in the serum of North Atlantic fin whales, whose testosterone increase was more than 4-fold during late summer (August–September) compared to the concentrations of the early summer (June) (Kjeld *et al.*, 1992). Nevertheless, that migratory population showed this rise in the summer feeding grounds, several months before the reproductive activity that takes place in winter (December–January). According to several studies, this increment in testosterone observed in migratory individuals, while they are still in their feeding areas at high latitudes, could suggest a physiological preparation before reaching the reproductive areas (Kjeld *et al.*, 2004; Vu *et al.*, 2015). In our study, little data prior to summer (May–June) prevented the detection of such a preparation stage. Nevertheless, the observation of two courtship displays in August and September may discard the scenario of a long preparatory phase in the males of the Gulf of California population and rather suggests the start of the mating season during the late summer. Since the mating season generally occurs over several months (Evans, 1987), it is possible that testosterone concentrations keep increasing during the autumn. Due to the lack of samples in autumn, and the unknown time lag in detection of these hormones in the blubber, we could not determine the precise range of the breeding season. Nevertheless, low values of testosterone in winter (Fig. 3, panel A) suggest the termination of mating occurs before this season.

Based on the evidence found in our study and what has been described about the gestation and lactation durations in other fin whale populations (Lockyer, 1984; Mizroch *et al.*, 1984), we propose a hypothetical reproductive cycle for the resident fin whale population in the Gulf of California (Fig. 3, panel B). Mating activity would occur during the late summer/autumn, part of gestation and lactation would take place around winter/spring, and weaning would follow during summer/autumn, depending on when conception occurs.

In marine and terrestrial mammals, a seasonal reproductive cycle implies a limited period for giving birth, usually as an adaptation to changes in the environment (McGuire *et al.*, 2010). In migratory whale species, the calving season seems to be driven partially by the necessity to avoid thermal stress for the newborns, giving birth in warm, low-latitude waters (Gaskin, 1982). Comparing to high latitudes, the sea surface temperature in the Gulf of California in winter is relatively warm (20.37 ± 1.92) (Escalante *et al.*, 2013). In fact, during this season, the Gulf and surroundings are used as an important calving area by several migratory whale species, such as the gray whale (Rice *et al.*, 1982), the humpback whale (Urbán and Aguayo, 1987), and the blue whale (Gendron and Ugalde De La Cruz, 2012). This led us to discard temperature stress as a driving factor of the seasonal reproductive cycle for the fin whale in the Gulf of California.

Seasonal reproductive strategies have also been observed among animals that live in more stable temperature conditions (Clutton-Brock and Harvey, 1978). In these cases, food availability may be an important limiting factor for any biological activity related to reproduction. As reported for the resident fin whales of the Mediterranean Sea (Relini *et al.*, 1992; Canese *et al.*, 2006), those in the Gulf of California are observed feeding all year (Ladrón de Guevara *et al.*, 2008). However, their main prey in the gulf is the euphausiid *Nyctiphanes simplex* (Tershy, 1992), which is most abundant in winter/spring and decreases in summer (Brinton and Townsend, 1980), when they shift their diet to small pelagic fish (Tershy, 1992; Gendron *et al.*, 2001). Thus, although this population does not fast as other migratory populations do, resources may not be enough year-round (in terms of prey abundance or nutritional quality) to support the strong energetic demands of pregnant and lactating females, for which the energetic cost can double that of the gestation and fetal development period (Lockyer, 1981). In most migratory populations of mysticetes, females fast or feed infrequently during lactation, often showing poor body condition (Aguilar and Borrell, 1990), and relying on the energy reserves stored during the feeding season. Nevertheless, in the Gulf of California, most females with calves appear in good body condition (D. Gendron, unpublished data), suggesting that they do not fast during lactation. Based on our results, it is likely that early- or mid-lactation, when calves are completely dependent on the mother’s milk (Oftedal, 1997), would start slightly before the most productive period of the year, that is the winter/spring (Álvarez-Borrego and Lara-Lara, 1991). Thus, the lactation period for the female C003 probably began in autumn, and therefore the weaning would have taken place during the following summer/autumn after we observed her with a calf. This is supported by the sighting of the female C021 with her calf in late summer (August), and its posterior re-sighting 6 months later during winter (February) without the calf (Fig. 3, panel B). This is analogous to the pattern of migratory rorquals, whose calves are weaned just before or immediately after the female’s arrival to the feeding grounds (Oftedal, 1997). All these observations support the hypothesis that prey availability or quality could be crucial in the regulation of the reproductive cycle of the resident fin whale population of the Gulf of California.

Reproduction responds to a complex interaction between hypothalamic–pituitary–gonad axis and environmental factors (Nelson *et al.*, 1990). A short reproductive period in a restricted area makes this population more vulnerable to environmental changes and anthropogenic disturbances. In fact, changes in survival rate of the calves (Lockyer, 1984), prey availability (Lockyer, 1986), or fertility (Hohn *et al.*, 2007) (e.g. for exposure to contaminants) may negatively affect the reproductive rate of a population and more so if it does not breed continually during the year. This also highlights the importance of specific measures that should be taken into account in the conservation plan of this population.

## Conclusions

Progesterone and testosterone assays were validated for the first time in free-ranging fin whales. Our study provides preliminary baseline parameters for reproductive hormones in fin whale blubber that, in the future, will allow the monitoring of their variations. In particular, the separation of two clusters in progesterone concentration lays the basis for the future determination of different reproductive categories of females. Furthermore, contrary to what has been observed in other resident mysticete populations, here we report a seasonal reproductive cycle for the fin whale population of the Gulf of California, which provides a useful tool for estimating other reproductive parameters for its management. In order to get a more precise insight into its reproductive dynamics, future studies need to incorporate photogrammetry analyses to account for different stages of individual development at varying hormone concentrations, to increase the time series monitoring to detect more details of the seasonal cycle, and to estimate possible inter-annual anomalies from it.

## Supplementary Material

carone_etal_2019_supplementary_material_coz059
