# Supplementary material for: Sex steroid hormones and behavior reveal seasonal reproduction in a resident fin whale population
Source: Conserv Physiol. 2019 Oct 31;7(1):coz059. doi: 10.1093/conphys/coz059 (PMC6822541; doi:10.1093/conphys/coz059)
Supplement: carone_etal_2019_supplementary_material_coz059 [file carone_etal_2019_supplementary_material_coz059.pdf]

Supplementary Materials:

Sex steroid hormones and behavior reveal seasonal reproduction in a resident fin whale population

Erica Carone[1], Mario A. Pardo[2], Shannon Atkinson[3], Kendall Mashburn[3], Héctor Pérez-Puig[4], Luis Enríquez-Paredes[5], Diane Gendron[1,*]

[1] Centro Interdisciplinario de Ciencias Marinas, Instituto Politécnico Nacional. La Paz, Baja California Sur 23096, Mexico. Email: erica83.cami@gmail.com

[2] CONACYT - Centro de Investigación Científica y de Educación Superior de Ensenada, Unidad La Paz. La Paz, Baja California Sur 23050, Mexico. Email: mpardo@cicese.mx

[3] University of Alaska Fairbanks, College of Fisheries and Ocean Sciences. Juneau AK 99801, USA. Email: skatkinson@alaska.edu & klmashburn@alaska.edu

[4] Kino Bay Center for Cultural and Ecological Studies, Prescott College. Bahía de Kino, Sonora 83340, Mexico. Email: hector.perez@prescott.edu

[5] Facultad de Ciencias Marinas, Universidad Autónoma de Baja California, Ensenada, Baja California 22800 Mexico. Email: lmenriquez@uabc.edu.mx

**Corresponding author:* dianegendroncicimar@gmail.com, Phone (52+612)1225366 ext 81516

Supplementary Text 1. Mixture model of progesterone concentrations.

Apparent bimodal pattern of progesterone concentrations :

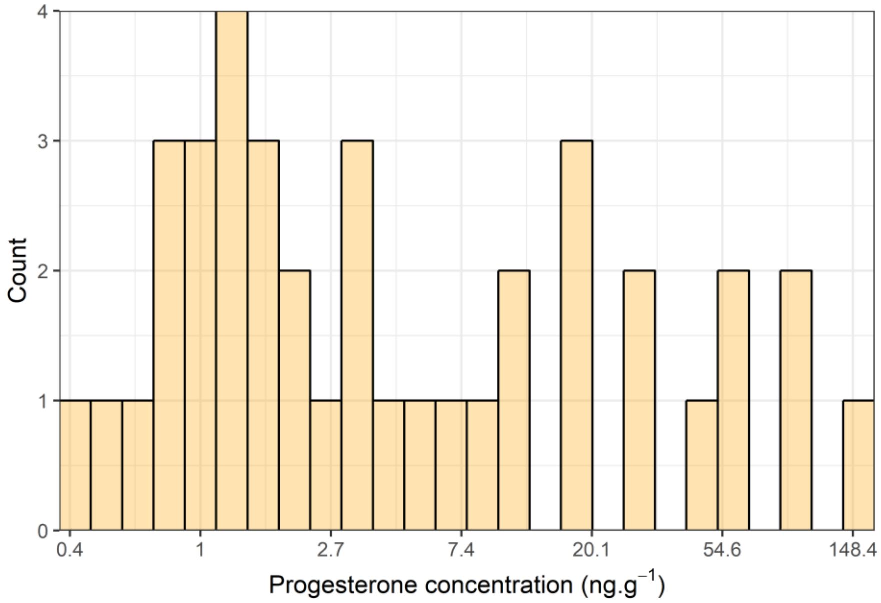

Algebraic representation:

The logarithmic scale of progesterone concentrations (*H*) at each biopsy (*i*) are stated as coming from a normal distribution:

$$\log(H_i) \sim N(\mu_{H_i}, \tau_{H_i})$$

Its stochastic means (μ) and precisions (τ) are grouped in a known number of clusters (*C*), with fixed effects . A half-normal likelihood was stated for the means, with a broad standard deviation, and an uninformative distribution for the precision:

$$\mu_{P_i} = \tilde{\mu}_{C_i} \;;\quad \tau_{P_i} = \tilde{\tau}_{C_i}$$

The clusters have a categorical distribution; whose stochastic parameter represents the probability of the source for each observation (*i*) of each cluster (*C*):

$$C_i \sim Cat(p_C)$$

The prior for such probability of cluster source (*p*) was a Dirichlet for both clusters:

$$p_C \sim Dir(C)$$

JAGS code:

```
"model {

 # We have hormone log-progesterone values
 # that we suppose are generated by a mixture
 # of two different normal distributions (i.e. clusters).
 # We don't know which datum came from each cluster.
 # Our goal is to estimate the probability that each
 # score came from each of the two clusters,
# That is, an ordered vector of the posterior values of
 # p_cluster[1] and p_cluster[2]. We are also interested
# in the means and SDs of the normal distributions
 # that describe those clusters, and the probability of
 # occurrence of each cluster, which are simply the
 # posteriors of the same parameter (p_cluster).

 for (i in 1:n_prog) {
   log_prog[i] ~ dnorm(mu_log_prog[i], tau_log_prog[i])   # Mixture of normal
   mu_log_prog[i] <- mu_cluster[cluster[i]]               # Fixed means per cluster
   tau_log_prog[i] <- tau_cluster[cluster[i]]             # Fixed precision per cluster
   cluster[i] ~ dcat(p_cluster[1:n_cluster])              # Categorical likelihood
 }
 # Fixed effects on clusters:
 for (j in 1:n_cluster) {
   mu_cluster[j] ~ dnorm(0, 1.0E-10)                      # Half-normal
   tau_cluster[j] ~ dunif(0.2, 2.1)                       # Uniform
   sd_cluster[j] <- sqrt(1/tau_cluster[j])                # Defines SDs from precision
 }
 # Priors:
 p_cluster[1:n_cluster] ~ ddirch(ones_rep_n_cluster)      # The Dirichlet prior

}"
```

Supplementary Figure 1. Parallelism test

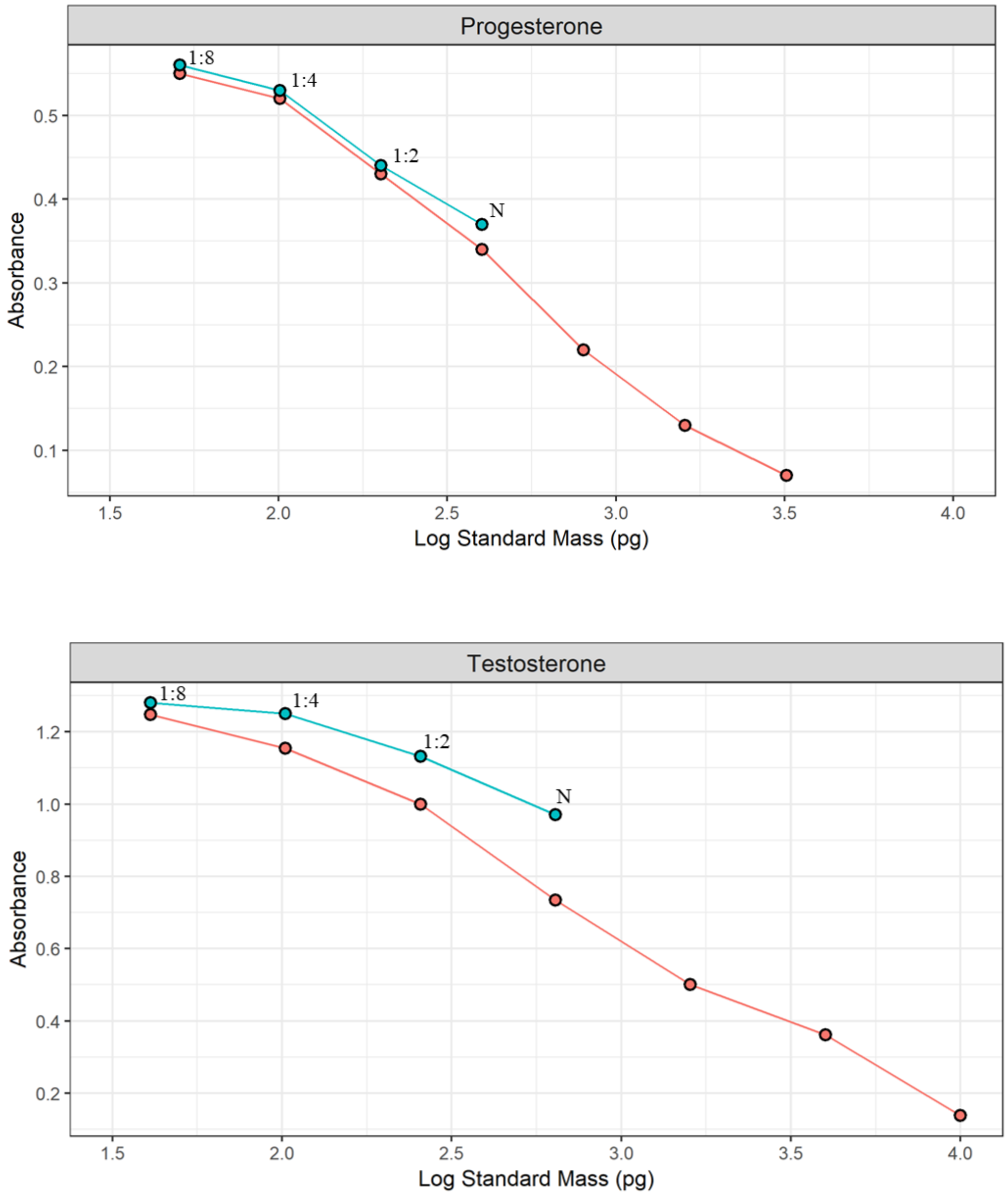

Supplementary Figure 1. Serial dilutions of samples show parallelism with the standards of progesterone and testosterone. The standard curves are indicated by the orange circles, while the blubber dilutions are indicated by green circles.

Supplementary Figure 2. Accuracy test

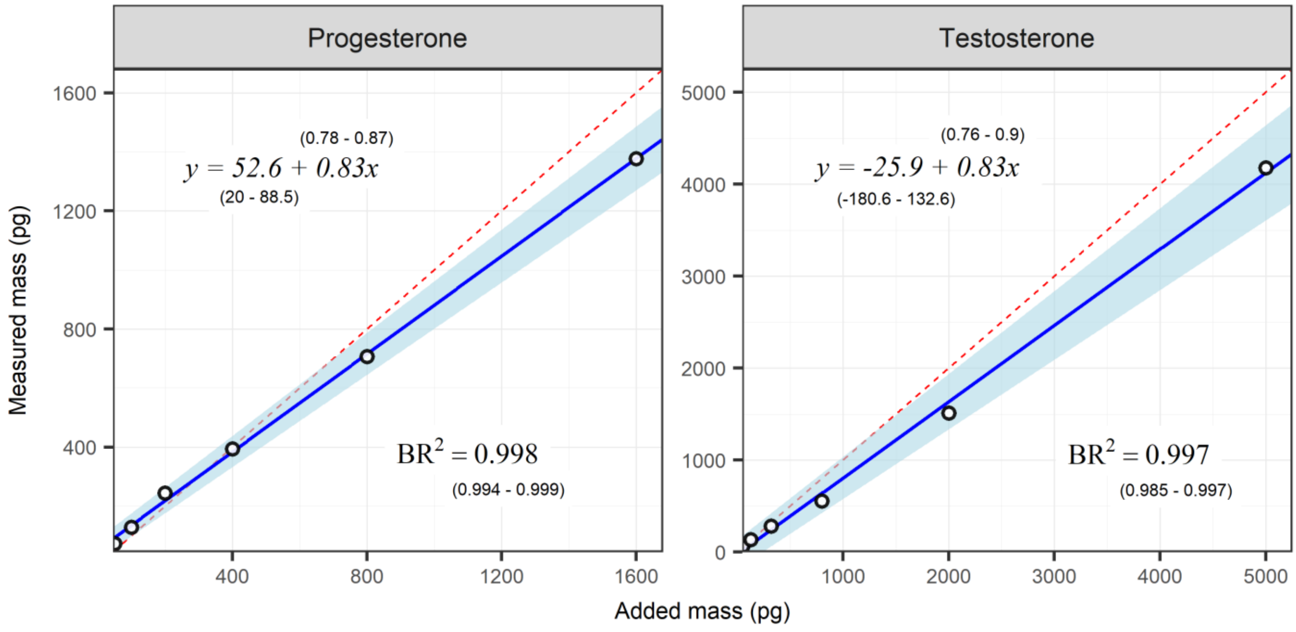

Supplementary Figure 2. Accuracy test to compare the slope of measured vs. added (i.e. theoretical) masses of progesterone (left) and testosterone (right) in fin whales blubber. The posterior median of the regression coefficients are in the main equation (blue tick line). The values in parenthesis close to them represent the 95%-credible intervals (blue shaded area), as well as those of the Bayesian R-squared (BR^2). The dashed red line represents the hypothetical 1:1 ratio.

Supplementary Table 1

Supplementary Table 1. Original dataset of hormone concentrations reported as ng g^{-1} of blubber extracted. In the columns are reported also the gender and date.

Gender	Day	Month	Year	Hormone	Concentration
**Female**	16	3	2007	progesterone	63
**Female**	16	3	2007	progesterone	17.88
**Female**	18	3	2007	progesterone	31.66
**Female**	19	4	2007	progesterone	9.98
**Female**	9	2	2008	progesterone	44.21
**Female**	11	2	2008	progesterone	1.09
**Female**	11	2	2008	progesterone	0.61
**Female**	23	3	2008	progesterone	1.41
**Female**	23	4	2009	progesterone	3.35
**Female**	23	4	2009	progesterone	1.03
**Female**	23	4	2009	progesterone	2.84
**Female**	24	4	2009	progesterone	6.90
**Female**	24	4	2009	progesterone	25.65
**Female**	24	4	2009	progesterone	1.70
**Female**	24	4	2009	progesterone	4.08
**Female**	20	3	2017	progesterone	1.92
**Female**	20	3	2017	progesterone	1.67
**Female**	6	8	2015	progesterone	173.36
**Female**	7	8	2015	progesterone	2.06
**Female**	7	8	2015	progesterone	3.47
**Female**	12	8	2015	progesterone	87.07
**Female**	12	8	2015	progesterone	18.26
**Female**	12	8	2015	progesterone	12.43
**Female**	17	7	2016	progesterone	55.20
**Female**	17	2	2016	progesterone	0.76
**Female**	19	2	2016	progesterone	0.93
**Female**	27	2	2016	progesterone	1.25
**Female**	27	2	2016	progesterone	1.36

**Female**	23	7	2016	progesterone	16.41
**Female**	23	7	2016	progesterone	1.13
**Female**	23	7	2016	progesterone	0.79
**Female**	24	7	2016	progesterone	1.67
**Female**	24	7	2016	progesterone	93.75
**Female**	26	7	2016	progesterone	5.81
**Female**	24	7	2016	progesterone	3.50
**Female**	24	7	2016	progesterone	0.85
**Female**	25	7	2016	progesterone	0.52
**Female**	25	7	2016	progesterone	8.10
**Female**	26	7	2016	progesterone	0.42
**Male**	18	3	2016	testosterone	1.18
**Male**	8	5	2007	testosterone	0.88
**Male**	8	5	2007	testosterone	0.45
**Male**	9	5	2007	testosterone	0.10
**Male**	9	5	2007	testosterone	0.52
**Male**	23	5	2007	testosterone	1.27
**Male**	23	3	2008	testosterone	4.60
**Male**	23	3	2008	testosterone	0.16
**Male**	14	3	2009	testosterone	1.25
**Male**	29	3	2009	testosterone	0.06
**Male**	21	4	2009	testosterone	0.31
**Male**	23	4	2009	testosterone	0.35
**Male**	23	4	2009	testosterone	1.03
**Male**	23	4	2009	testosterone	1.29
**Male**	23	4	2009	testosterone	1.02
**Male**	23	4	2009	testosterone	0.50
**Male**	23	4	2009	testosterone	0.62
**Male**	24	4	2009	testosterone	1.89
**Male**	24	4	2009	testosterone	0.33
**Male**	20	3	2017	testosterone	1.64
**Male**	20	3	2017	testosterone	0.37
**Male**	20	3	2017	testosterone	0.41
**Male**	20	3	2017	testosterone	1.11

**Male**	6	8	2015	testosterone	9.21
**Male**	6	8	2015	testosterone	6
**Male**	9	8	2015	testosterone	1.88
**Male**	12	8	2015	testosterone	7.20
**Male**	12	8	2015	testosterone	7.86
**Male**	12	8	2015	testosterone	6.32
**Male**	12	8	2015	testosterone	3.58
**Male**	13	8	2015	testosterone	14.22
**Male**	12	2	2016	testosterone	0.20
**Male**	17	2	2016	testosterone	0.70
**Male**	17	2	2016	testosterone	0.05
**Male**	19	2	2016	testosterone	1.95
**Male**	27	2	2016	testosterone	0.21
**Male**	27	2	2016	testosterone	0.15
**Male**	27	2	2016	testosterone	0.18
**Male**	23	7	2016	testosterone	1.78
**Male**	23	7	2016	testosterone	0.94
**Male**	23	7	2016	testosterone	1.23
**Male**	24	7	2016	testosterone	0.47
**Male**	25	7	2016	testosterone	0.89
**Male**	26	7	2016	testosterone	0.35
**Male**	26	7	2016	testosterone	3.54

Supplementary Video 1

On September 17, 2018, we observed for the second time courtship behavior in the Ballenas Channel (Gulf of California). The sex of the animals involved was determined genetically. A sighting of a female (individual A) and a male (individual B) started at 8:19 am. The couple were surfacing in synchrony one after the other. The female was always leading, and the male was following. Both animals were swimming calmly while surfacing, but without a clear direction or pattern. At 11:37 am, a second male (individual C) joined the pair. Since that moment, the behavior of the group changed drastically: the males started to chase the female. We were able to drone filming for some minutes, during which one of the males performed a strong lateral stroke with its fluke towards the other male’s body, immediately after leaving the surface for a vertical dive. The female was always being followed, keeping a variable distance from the males, and sometimes changing direction abruptly.

At 12:46 pm, the female left the group and we lost track of her. After that, the surface behavior of the two males became more calm. Although both kept surfacing close to each other in synchrony, B leading and C following, the force of their surfacing was normal. The observation ended at 1:22 pm because of weather conditions.
